# Divergent pathogenic strategies of *Fusarium* species in *Panax notoginseng* and biocontrol by a *Bacillus-Serratia* consortium

**DOI:** 10.1186/s12870-026-08617-4

**Published:** 2026-03-24

**Authors:** Yinglong Deng, Qiongying Kang, Bichen Yang, Yuxuan Wang, Yongqi Zhou, Pili Yu, Zhiyuan Tong, Jinbao Dong, Xiaoting Huang, Wentao Wu, Jiaqing Wu, Youyong Zhu, Xiahong He, Liwei Guo

**Affiliations:** 1 State Key Laboratory for Conservation and Utilization of Bio-Resources in Yunnan, College of Plant Protection, Yunnan Agricultural University, Kunming, 650201 China; 2 Yunnan Provincial Key Laboratory for Conservation and Utilization of In-forest Resource, Southwest Forestry University, Kunming, Yunnan 650224 China; 3 Key Laboratory for Forest Resources Conservation and Utilization in the Southwest Mountains of China, Ministry of Education, Southwest Forestry University, Kunming, Yunnan 650224 China

**Keywords:** Pathogenicity, *Fusarium* spp., *Panax notoginseng*, Root rot, *Bacillus subtilis*, *Serratia marcesc*ens, Induced systemic resistance

## Abstract

*Fusarium*-induced root rot severely constrains *Panax notoginseng* cultivation, yet the differential pathogenicity and ecological interactions among coexisting *Fusarium* species remain poorly understood. By isolating three pathogenic isolates—*Fusarium oxysporum* LP1 and *F. solani* LP2/LP3—we uncovered a distinct ecological trade-off: *F. oxysporum* exhibits higher niche occupancy, whereas *F. solani* demonstrates markedly greater virulence.

To manage this disease, we engineered the *Bacillus subtilis-Serratia marcescens* synthetic microbial consortium (BS) comprising antagonistic strains XY-6 and XB-7. BS application significantly enhanced disease suppression (56%) compared with single-strain treatments (21%–25%). Mechanistic evaluations revealed a synergistic triple-mode of action: (i) direct pathogen inhibition via cell-free filtrates (38% reduction); (ii) activation of host systemic resistance, evidenced by substantially increased peroxidase (133%) and superoxide dismutase (173%) activities; and (iii) beneficial restructuring of the rhizosphere fungal community, characterized by *Mortierellomycota* enrichment, elevated α-diversity, and fortified microbial interaction networks.

Collectively, this study illuminates the ecological trade-off between virulence and niche fitness among *Fusarium* species, and provides a robust, sustainable microbiome-engineering strategy for mitigating soil-borne diseases in medicinal crops.

**Supplementary Information:**

The online version contains supplementary material available at 10.1186/s12870-026-08617-4.

## Introduction

*Fusarium*-induced root rot, caused mainly by soil-dwelling fungal pathogens, poses a serious challenge to agricultural output and ecological balance worldwide [1–3]. A five-year study (2014–2018) conducted in Australian grain production areas found this disease affecting over half (52%) of cereal crops in northern zones, with slightly lower prevalence rates of 31% and 29% observed in western and southern regions respectively, based on an examination of 1,774 fields [4]. The infection damages root architecture, significantly reducing the plant’s capacity to absorb water and essential minerals, leading to substantial production declines in key agricultural regions [5]. The medicinal herb *Panax notoginseng* (Burk.) F.H. Chen (*Panax*, Araliaceae), renowned for its therapeutic compounds that aid blood clotting and pain relief [6], faces severe cultivation constraints due to *Fusarium* infections, with documented production decreases surpassing 70% in affected areas [7]. Although *F. oxysporum* and *F. solani* have been identified as the dominant pathogens in this system, an important question remains whether these fungal species demonstrate distinct survival mechanisms in their host interactions. Understanding whether niche dominance (characterized by high population density within a specific ecological niche) and pathogenic advantage (manifested through elevated virulence) represent opposing biological strategies is essential for designing precise intervention methods.

Current research has extensively documented virulence factors in major crops like maize, wheat, and bananas [8–10], yet the pathogenic mechanisms of *Fusarium* species in *P. notoginseng* remain poorly understood. Studies have shown that the pathogenic process of *Fusarium* is initiated by extracellular enzymes that breach the plant cell wall. Enzymes such as pectinases, chitinases, cellulases, and hemicellulases degrade essential structural components of plant tissues [11]. Notably, various *Fusarium* species exhibit divergent infection strategies [12]. Determining whether these pathogens engage in competitive or cooperative interactions, along with clarifying their distinct virulence mechanisms, is vital for formulating species-specific management approaches.

Concurrently, the plant-associated microbiome constitutes an extended immune system, functioning as the first line of defense against soil-borne pathogens [13, 14]. Microorganisms that confer benefits bolster plant defenses through multiple mechanisms, including competitive exclusion, antimicrobial activity, and the activation of systemic resistance pathways [4, 15, 16]. Research has demonstrated that certain microbial strains such as *Pseudomonas*, *Variovorax*, and *Cladosporium* species found in the rhizosphere of *P. notoginseng* exhibit inhibitory effects against *F. solani* [17]. These findings have generated significant interest in synthetic microbial communities (SynComs): carefully engineered microbial assemblages that utilize interspecies relationships to enhance crop protection with improved reliability and performance compared to individual microbial applications [18]. SynComs demonstrate superior functional consistency and effectiveness relative to single-strain treatments, positioning them as valuable solutions for managing soil-borne plant diseases [19]. While these engineered communities have shown success in combating *Fusarium* wilt in various agricultural systems [20–22], their potential for controlling *Fusarium*-induced root rot in *P. notoginseng* remains further investigation.

The therapeutic potential of *P. notoginseng* has yet to be fully investigated. Although *Bacillus* species demonstrate wide-ranging antifungal properties, their individual effectiveness is frequently constrained by poor root zone establishment and survival stability [23]. Contemporary research has adopted an innovative approach by developing microbial consortia containing *Bacillus* strains, where synergistic interactions with complementary microorganisms can improve functional outcomes [19, 24]. Documented examples include successful combinations with *Pseudomonas* [24] or *Enterobacter* [25] species. *Serratia marcescens* emerges as another viable component for consortium development, given its rhizospheric presence and capacity to synthesize chitin-degrading enzymes along with various antifungal metabolites [26]. However, comprehensive studies examining collaborative effects between *Bacillus* and *Serratia* for plant protection applications remain notably absent. This knowledge gap presents a valuable prospect for creating an innovative microbial formulation with enhanced biocontrol capabilities.

The current investigation pursues three primary objectives: (1) to conduct a comprehensive assessment of virulence characteristics and ecological specialization among dominant *Fusarium* pathogens linked to root rot in *P. notoginseng*; (2) to design and validate an innovative microbial consortium combining *Bacillus* and *Serratia* strains for biological control applications; (3) to decode the multi-layered suppression mechanisms employed by this synthetic community, with particular emphasis on three key aspects: direct pathogen inhibition, activation of plant defense responses, and functional restructuring of rhizosphere microbial communities.

## Materials and methods

### Plant material and ethical statement

Seeds and seedlings of *P. notoginseng* (Burkill) F.H.Chen (common name: Sanqi) used in this study were commercially obtained as disease-free material from Lüfeng Agricultural By-Products Management Department in Wenshan City, Yunnan Province, China. Wenshan is the authentic production area of this species. The seeds were morphologically identified by the authors. This study did not involve endangered or protected species, and the use of commercially sourced plant materials complied with all relevant institutional and national guidelines. No specific permissions were required.

### Isolation and morphological identification of potential pathogens

Soil samples were collected from a *P. notoginseng* plantation under a pine forest with severe root rot symptoms in Laotanshan Village (Lancang County, Yunnan Province, China). Fungal isolation was performed using the soil dilution plate method: 10 g of soil was suspended in 90 mL sterile water, shaken at 120 rpm for 90 min, serially diluted, and 50 µL of the 10^− 5^ dilution was plated on PDA. Plates were incubated at 25 °C in darkness for 5 days. The pure isolates were preliminarily characterized based on morphological traits on different culture media. Each isolate was point-inoculated onto plates of potato dextrose agar (PDA) [27], synthetic nutrient agar (SNA), maize meal agar (MMA) [28], Czapek medium, and oatmeal agar (OA) [29]. The cultures were incubated at 25 ℃ under a 12-h light/12-h dark cycle for 7 days. Colony characteristics and microscopic features of conidia were examined.

### Molecular identification and phylogenetic analysis

Genomic DNA was extracted from fresh mycelia of pure isolates using a commercial fungal genomic DNA extraction kit (Aidlab Biotechnologies Co., Ltd., Beijing, China) following the manufacturer’s instructions. Three genetic loci—the internal transcribed spacer (ITS) region of the ribosomal DNA, the partial large subunit (LSU) rRNA gene, and the translation elongation factor 1-alpha (TEF-1α) gene—were amplified by polymerase chain reaction (PCR) for species identification. The primers were used: ITS1F(5′-CTTGGTCATAGGAAGAAGTAA-3′)/ITS4R(5′-TCTCGCTTATGATATGC-3′) for ITS; LR0Rf(5′-GTACCCGCTGAACTTAAGC-3′)/LR5r(5′-ATCCTGAGGGAAACTTC-3′) for LSU; EF1-728 F(5′-CATCGAGAAGTTCGAGAAGG-3′)/EF2(5′-GGARGTACCAGTSATCATGTT-3′) for TEF-1α.

Each 50-µL PCR reaction consisted of 45 µL of 1× TSE101 Gold Master Mix (Tsingke, Beijing, China), 2 µL of each primer (0.4 µM), and 1 µL of genomic DNA at a concentration of 50 ng/µL. The thermal cycling conditions were as follows: an initial denaturation at 95 °C for 2 min; followed by 35 cycles of denaturation at 95 °C for 30 s, annealing at 55 °C for 30 s, and extension at 72 °C for 1 min; and a final extension at 72 °C for 10 min. All PCR products were purified and sequenced in both directions by Tsingke Biotech (Beijing, China). The obtained sequences were queried against the NCBI GenBank nucleotide database using BLAST, and a phylogenetic tree was constructed using the neighbor-joining method in MEGA-X software [30].

### Pathogenicity assessment of *Fusarium*

#### In vitro pathogenicity assay on detached roots

Detached *P. notoginseng* roots were surface-sterilized (75% ethanol, 30 s; 1% sodium hypochlorite, 2 min) and subjected to two treatments: (i) wounding at the midpoint with a sterile scalpel, or (ii) no wounding. A 5-mm mycelial plug from the actively growing margin of a *Fusarium* isolate PDA culture was placed onto the inoculation site. Control root segments received sterile PDA plugs. All inoculated root segments were incubated in sterile Petri dishes with moist filter paper at 28 °C in the dark for 7 days, and lesion development was monitored daily. To fulfill Koch’s postulates, tissue from lesion margins was re-isolated on PDA at 25 °C and compared morphologically with the original inoculum [31].

#### Microscopic observation of *Fusarium* infection in *P. notoginseng*

Fungal colonization and infection structures were visualized using a dual-staining protocol with methyl blue and solophenyl flavine, followed by fluorescence microscopy. Each root segment was surface-sterilized and then inoculated at the midpoint with a 5-mm mycelial plug obtained from the growing margin of *Fusarium* isolates cultured on PDA. Following inoculation, the root segments were maintained in humidified Petri dishes at 28 °C for 7 days.

For staining, infected roots were first cleared in bleaching solution at 60 °C for 2 h, transferred to preheated ddH₂O, and stored at 4 °C for 24 h. The cleared tissues were subsequently stained with a methyl blue-solophenyl flavine mixture (≥ 5 mL) for 30 min. Following rinsing, samples were mounted in 1% hydrochloric acid and covered with a coverslip. Fungal structures were observed under a fluorescence microscope with 470 nm excitation [32].

#### Pot-based pathogenicity assay under controlled conditions

Pathogenicity was further quantified in a pot experiment under controlled environmental conditions. The inoculum of each *Fusarium* isolate was prepared by flooding 10-day-old PDA cultures with sterile distilled water, gently scraping the surface to release spores. The spore concentration was determined using a hemocytometer and adjusted to 1 × 10⁵, 1 × 10⁶, and 1 × 10⁷ spores mL⁻¹ with sterile water [7].

*P. notoginseng* seedlings were transplanted into tissue culture bottles containing 20 g of sterile forest soil with no prior history of *P. notoginseng* cultivation. Ten seedlings were planted per bottle, and each seedling received a 10 mL rhizosphere drench of the spore suspension. Control plants were treated with an equal volume of sterile water. Each treatment was performed in three replicates. All bottles were arranged randomly in a growth incubator maintained at 28 °C and 80% relative humidity. Disease incidence was assessed three months post-inoculation and calculated as follows:

1 $$\begin{array}{ll}\text{Incidence rate}\,(\%)=\\\left(\text{number of infected plants}/\text{total number of inoculated plants}\right)\\ \times 100\end{array}$$

### Cell wall-degrading enzyme (CWDE) activity analysis

#### Effect of crude *Fusarium* CWDE extracts on root pathogenicity

CWDE extracts were prepared from *Fusarium* species cultured in Czapek-based liquid induction media. The basal medium contained (per liter) 2 g KNO₃, 0.5 g KCl, 0.01 g FeSO₄, 1.0 g K₂HPO₄, 0.5 g MgSO₄·7 H₂O, and 10 g of specific carbon source, adjusted to pH 5.0. The same weight of bran and sodium carboxymethyl cellulose (CMCNa) was substituted for pectin to obtain different induction media. Mycelial plugs (5 mm diameter) from actively growing colony margins were inoculated into 150 mL of enzyme production medium (six plugs per flask) and incubated at 28 °C with continuous shaking (120 rpm) for 7 days. Culture filtrates were sequentially filtered through four layers of sterile gauze and centrifuged at 10,000 × g for 15 min at 4 °C. The resulting supernatants were collected as crude enzyme solutions.

Pathogenicity assays were performed by applying 20 µL of crude enzyme solution onto surface-sterilized root segments in Petri dishes. Control treatments were amended with sterile culture medium. After 7 days of incubation under moist conditions at room temperature, disease incidence was recorded. The experiment consisted of three independent biological replicates per treatment [33].

#### Assay of different CWDE activities in *Fusarium*

To evaluate the activity of CWDEs produced by *Fusarium* in *P. notoginseng*, surface-sterilized *P. notoginseng* roots were bisected and inoculated with mycelial plugs of *Fusarium* isolates at the incision site. After 5 d of incubation at 28 °C, infected tissues were homogenized in extraction buffer under ice-cold conditions. The homogenate was centrifuged at 10,000 rpm for 20 min at 4 °C, and the supernatant was filter-sterilized through a 0.22 μm membrane to obtain crude enzyme extracts. The activities of cellulases, including carboxymethyl cellulase (Cx) and β-glucosidase (β-GLU), and pectinases, including polygalacturonase (PG) and polymethylgalacturonase (PMG), in *P. notoginseng* roots infected with *Fusarium* were measured according to the method described by Cui et al. [34].

### Quantification of *F. oxysporum* and *F. solani* biomass across plant-soil niches

DNA was extracted from root tissues at different disease severity stages, as well as from roots, rhizosphere soil, and bulk soil of both healthy and diseased *P. notoginseng* plants. All samples were immediately flash-frozen in liquid nitrogen and stored at -80 °C until analysis. Total genomic DNA was extracted using the Aidlab FastBeat Soil DNA Kit (Aidlab Biotechnologies, Beijing, China) according to the manufacturer’s instructions.

Species-specific primer sets were employed for amplification: For *F. oxysporum*, the primers used were Fo-QF (5’-CTCAAACAGTGGTACATGCGAGG-3’) and Fo-QR (5’-CATCTAGGTCTTCCATCCACTTGA-3’); for *F. solani*, the primers Fs-QF (5’-CCACGCTTGTGAGCTATGTAGTTC-3’) and Fs-QR (5’-CTCTTGAGGTAGACCACAGTAGGC-3’) were utilized [27, 35]. Each 20 µL qPCR reaction consisted of 10 µL of 2× SYBR Green Premix Pro Taq HS (Accurate Biology, China), 0.4 µM of each primer, 2 µL of DNA template, and nuclease-free water to 20 µL. Reactions were carried out on a LightCycler^®^ 96 Real-Time PCR System (Roche Diagnostics, Mannheim, Germany) with an initial denaturation at 95 ℃ for 2 min, followed by 45 cycles of 95 °C for 1 min, 62 °C for 30 s, and 72 °C for 1 min. Fluorescence was recorded during the extension step, and all reactions were performed in triplicate. For absolute quantification, standard curves were generated using serial dilutions of plasmid DNA containing the target sequences to enable absolute quantification of pathogen biomass in each sample type.

### Construction and evaluation of synthetic microbial communities (SynComs)

#### Isolation and antagonistic screening of biocontrol microorganisms

Bacterial isolates were obtained from the same soil samples as the Fusarium pathogens using the dilution plate method. Bacterial colonies with distinct morphological and pigmentation characteristics were selected for purification and pre-cultured in Luria-Bertani (LB) broth at 28 °C with shaking at 180 rpm for 24 h.

Antagonistic activity was assessed using a dual-culture assay. Mycelial plugs (5 mm) of F. oxysporum LP1 and F. solani LP2 were placed at the center of fresh PDA plates, with bacterial isolates inoculated at four symmetrical positions, 2.5 cm from the pathogen plug. Each treatment was repeated four times. After incubation at 28 °C for 7 days, pathogen growth inhibition was measured using the cross-method and calculated as follows:

2 $$\text{Inhibition rate}\,(\%)=\left[\left(D_c-D_t\right) / D_c\right]\times100$$

Where Dc and Dt represent the colony diameters in control and treatment groups, respectively.

#### Compatibility assessment of selected strains

Based on superior antagonistic performance, *Bacillus* sp. strain XY-6 was selected as the core strain for compatibility testing with other isolates. Two complementary methods were employed: cross-streaking method [36] and V-shaped antagonism assay [37]. Overnight cultures (OD₆₀₀ = 0.6) were prepared. In the cross-streaking assay, XY-6 and each test strain were streaked perpendicularly on LB agar. For the V-shaped method, 3 µL of each culture was spotted along two opposing oblique lines in a seven-point “V” pattern. Plates were incubated at 28 °C for 3 days, with strains considered compatible if no inhibition zones were observed at interaction sites.

#### SynComs assembly and greenhouse validation

Compatible strains were combined with XY-6 at a 1:1 (v/v) ratio to form SynComs after resuspension in sterile water (OD₆₀₀ = 0.6). The biocontrol efficacy was evaluated in greenhouse conditions using continuous cropping soil with two-year *P. notoginseng* history. Each pot containing 20 g of soil received 20 mL of a bacterial suspension (single strain or SynComs), with sterile water as control. After one week, surface-sterilized seeds were sown (10 seeds per pot) with seven replicates per treatment. Root rot incidence and seedling survival rate were assessed 6 months post-inoculation [38]:

3 $$\begin{array}{ll}\text{Root rot incidence}\,(\%)\\=\text{Number of diseased seedlings}/\text{Total seeds sown}\times100\end{array}$$

4 $$\begin{array}{ll}\text{Seedling survival rate}(\%)=\\\text{Number of surviving seedlings}/\text{Total seeds sown}\times100\end{array}$$

The most effective strains and SynComs were advanced for further mechanistic studies.

### Molecular identification and functional profiling of SynComs members

The nearly full-length 16S rRNA gene was amplified with universal bacterial primers 27 F (5′-AGAGTTTGATCCTGGCTCAG-3′) and 1492R (5′-GGTTACCTTGTTACGACTT-3′). Sequencing was performed by Tsingke Biotechnology Co., Ltd. (Beijing). Resulting sequences were assembled and aligned against the NCBI database via BLAST. Phylogenetic analysis was constructed using the neighbor-joining method in MEGA-X software.

Plant growth-promoting traits were evaluated for strains XA-2, XY-6, and XB-7. Isolates were spot-inoculated onto specific solid media to assess phosphate solubilization, siderophore production, cellulase, protease, and laccase activity, as well as nitrogen fixation and potassium solubilization capabilities. Following a 5-day incubation at 28 °C, functional positivity was assessed. Potential nitrogen fixation was indicated by robust growth on nitrogen-free medium, whereas the manifestation of other specific functions was confirmed by the presence of distinct transparent zones surrounding the colonies on their respective assay media [39, 40].

IAA was quantified using a colorimetric method [41]. Strains were grown in tryptophan-supplemented LB broth. After centrifugation, the supernatant was reacted with an equal volume of Salkowski reagent; the development of a pink color indicates the production of IAA.

### Assessment of plant growth promotion and induced resistance by effective SynComs

The preselected biocontrol strains and SynComs were further evaluated in two distinct soil types: sterilized forest soil (with no history of *P. notoginseng* cultivation) and continuous cropping soil (with a two-year history of *P. notoginseng*). Uniform one-year-old seedlings were transplanted into pots, each containing approximately 800 g of soil and 10 seedlings.

Sterilized soil was inoculated with a spore suspension (1 × 10⁷ spores mL^-1^) of *F. solani* LP2, the most virulent strain. Single-strain or SynCom suspensions (OD₆₀₀ = 0.6) were then applied at 80 mL/pot; controls received water. Continuous cropping soil received the same treatments without pathogen inoculation. All treatments were replicated six times.

Disease incidence and root biomass were recorded after three months. Plant samples were flash-frozen in liquid nitrogen and stored at -80℃ for subsequent biochemical analyses. Malondialdehyde (MDA) content, the activities of peroxidase (POD) and superoxide dismutase (SOD) in root tissues were determined using commercial assay kits (Suzhou Comin Biotechnology Co., Ltd.), according to manufacturer protocols. Each treatment conducted with three replicates.

For in vitro antagonism assays, cell-free filtrates were prepared from 4-day bacterial cultures via centrifugation and triple filtration (0.22 μm). The sterile filtrate of the SynComs was prepared by mixing sterile filtrates from individual bacterial strains at a 1:1 ratio. PDA was amended with 2.5% or 40% (v/v) of this SynCom filtrate prior to solidification. For the 100% concentration treatment, 200 µL of undiluted filtrate was spread evenly to achieve complete surface coverage. All plates were then center-inoculated with a mycelial plug of *F. solani* LP2 and incubated at 28 °C for 7 days, alongside control plates containing no filtrate. Each treatment was performed with three independent replicates. Colony diameters were measured with the cross method.

### Microbiome sequencing and community analysis of SynComs

To investigate the impact of biocontrol strains on the soil microbial community, rhizosphere soil samples were collected from treatments with the most effective SynCom (BS), individual strains B and S, and the control. Total genomic DNA was extracted using the DNeasy^®^ PowerSoil^®^ Pro Kit (Qiagen, USA). The fungal ITS region and bacterial 16S rRNA gene were amplified with barcoded primers ITS1F (CTTGGTCATTTAGAGGAAGTAA)/ITS2R (GCTGCGTTCTTCATCGATGC), and 338 F (ACTCCTACGGGAGGCAGCAG)/806R (GGACTACHVGGGTWTCTAAT), respectively. The resulting amplicons were purified and recovered. Sequencing libraries were constructed with the NEXTFLEX^®^ Rapid DNA‑Seq Kit (Bioo Scientific, USA) and paired‑end sequencing (2 × 300 bp) was performed on the Illumina MiSeq PE300 platform.

Raw sequencing data were processed using FLASH (v1.2.7) to merge paired‑end reads, and quality control was performed with fastp (v0.20.0). Operational Taxonomic Units (OTUs) were clustered at 97% similarity using the UPARSE algorithm within USEARCH (v7.1). Subsequent analyses including principal coordinate analysis (PCoA), microbial community composition, alpha diversity, and LEfSe (linear discriminant analysis effect size) were performed on the Majorbio Cloud Platform (https://cloud.majorbio.com). The co-occurrence network analysis, as well as the analysis and visualization of the Spearman correlation heatmap, were conducted using the OmicStudio tools platform (https://www.omicstudio.cn).

### Statistical analysis

Data were analysed using one-way ANOVA followed by Tukey’s post-hoc test or Student’s *t*-test where appropriate. Statistical significance was set at *P* < 0.05. All statistical analyses were conducted in IBM SPSS Statistics 18 (IBM Corp., Armonk, NY, USA). Figures were generated with GraphPad Prism 8.0 (GraphPad Software Inc., La Jolla, CA, USA).

## Results

### Revealing divergent pathogenic mechanisms within *Fusarium* species complex

#### Morphological and molecular identification of Fusarium strains

**Fig. 1:**
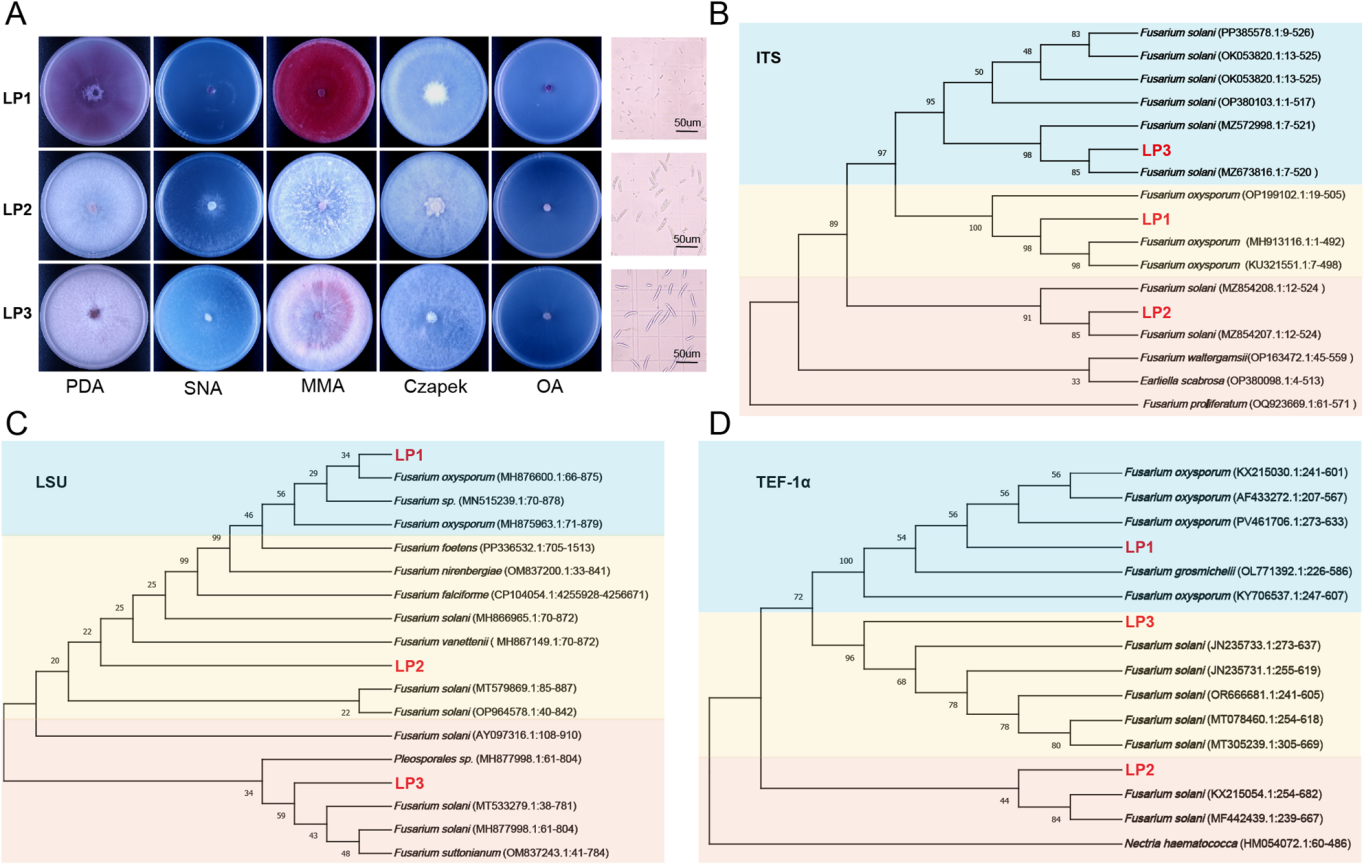
Morphological identification and phylogenetic tree construction of *Fusarium*. **A** Colony morphology of the pathogens on five media: potato dextrose agar (PDA), synthetic nutrient agar (SNA), maize meal agar (MMA), Czapek agar, and oatmeal agar (OA), as well as spore morphology on PDA; **B** Phylogenetic tree based on ITS; **C** Phylogenetic tree based on LSU; **D** Phylogenetic tree based on TEF-1α

A total of six *Fusarium* isolates were recovered from the soil. Based on their unique colony morphology observed across different growth media, three representative strains (LP1, LP2, and LP3) were chosen for comprehensive analysis (Fig. 1A). Examination of conidial structures under microscopy revealed features typical of *Fusarium* species, as documented in previous studies [42–44] (Fig. 1A). Genetic characterization through sequencing of ITS, LSU, and TEF-1α regions identified LP1 as *F. oxysporum*, while LP2 and LP3 were classified as *F. solani* (Fig. 1B-D). An interesting observation was made regarding LP2/LP3’s genetic profile: although their ITS and TEF-1α sequences grouped with known *F. solani* strains, their LSU sequences formed a separate lineage within the clade, indicating possible genetic diversity within the species. The initial isolation results showed a predominance of *F. oxysporum*, with four out of six isolates (67%) belonging to this species, compared to only two *F. solani* isolates.

#### Differences in pathogenicity exist among Fusarium species

**Fig. 2:**
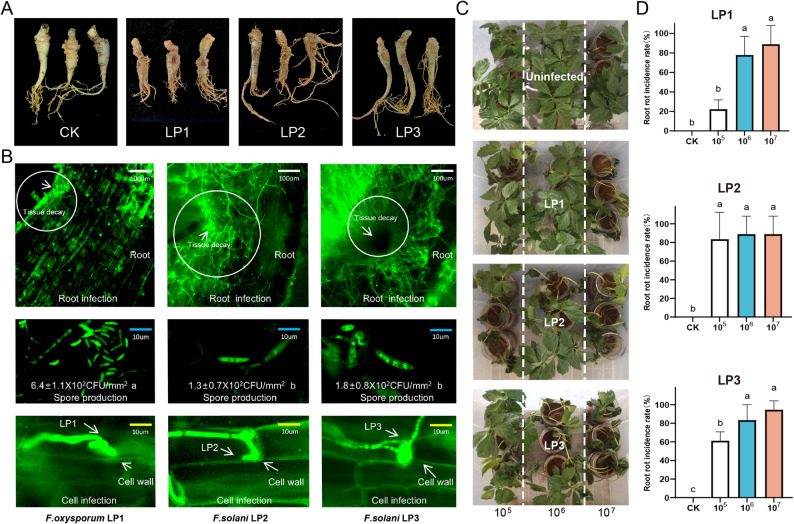
Pathogenicity assessment of *Fusarium* isolates. **A** Evaluation of pathogenicity of *Fusarium* strains inoculated on *P. notoginseng* with wounds in vitro; **B** Microscopic observation: *Fusarium* local infection, root surface spore count, and infection structures; **C, D** Effects of different *Fusarium* spore suspension concentrations on the incidence of root rot in *P. notoginseng*. a, b, and c indicate significant differences (*P* < 0.05)

The pathogenicity tests verified that all three *Fusarium* isolates were responsible for causing root rot, satisfying Koch’s postulates (Fig. 2A; Fig. S1). While these fungal strains demonstrated phosphorus solubilization capabilities on PSM medium, none exhibited siderophore production in CAS agar plate assays. Among the tested strains, LP2 and LP3 showed enzymatic activities on different media: both degraded cellulose on CCM agar and oxidized phenolic compounds on LCM agar. Notably, LP2 produced more extensive clear zones and larger colony diameters on LCM plates, indicating a higher level of laccase secretion compared with LP3 and LP1 (Fig. S2). These extracellular enzymes likely play significant roles in the development of root rot symptoms.

At the same inoculation time, LP2 and LP3 caused pronounced infection and tissue damage in *P. notoginseng* roots, with lesions occurring in localized regions. All three strains formed infection structures capable of penetrating and disrupting host cell walls, although their morphologies differed among strains. Notably, roots inoculated with LP1 exhibited a substantially higher abundance of *Fusarium* spores on the root surface than those inoculated with LP2 or LP3 (Fig. 2B).

Among the tested strains, *F. solani* LP2 exhibited the highest pathogenicity, causing disease in over 80% of the plants at the lowest inoculum dose. LP3 induced disease in approximately 60% of the plants, whereas *F. oxysporum* LP1 displayed markedly lower virulence, causing only 30% disease incidence under the same inoculum dose (Fig. 2C-D).

#### Ecological and mechanistic insights into the decoupling of Fusarium CWDE virulence and biomass accumulation

**Fig. 3:**
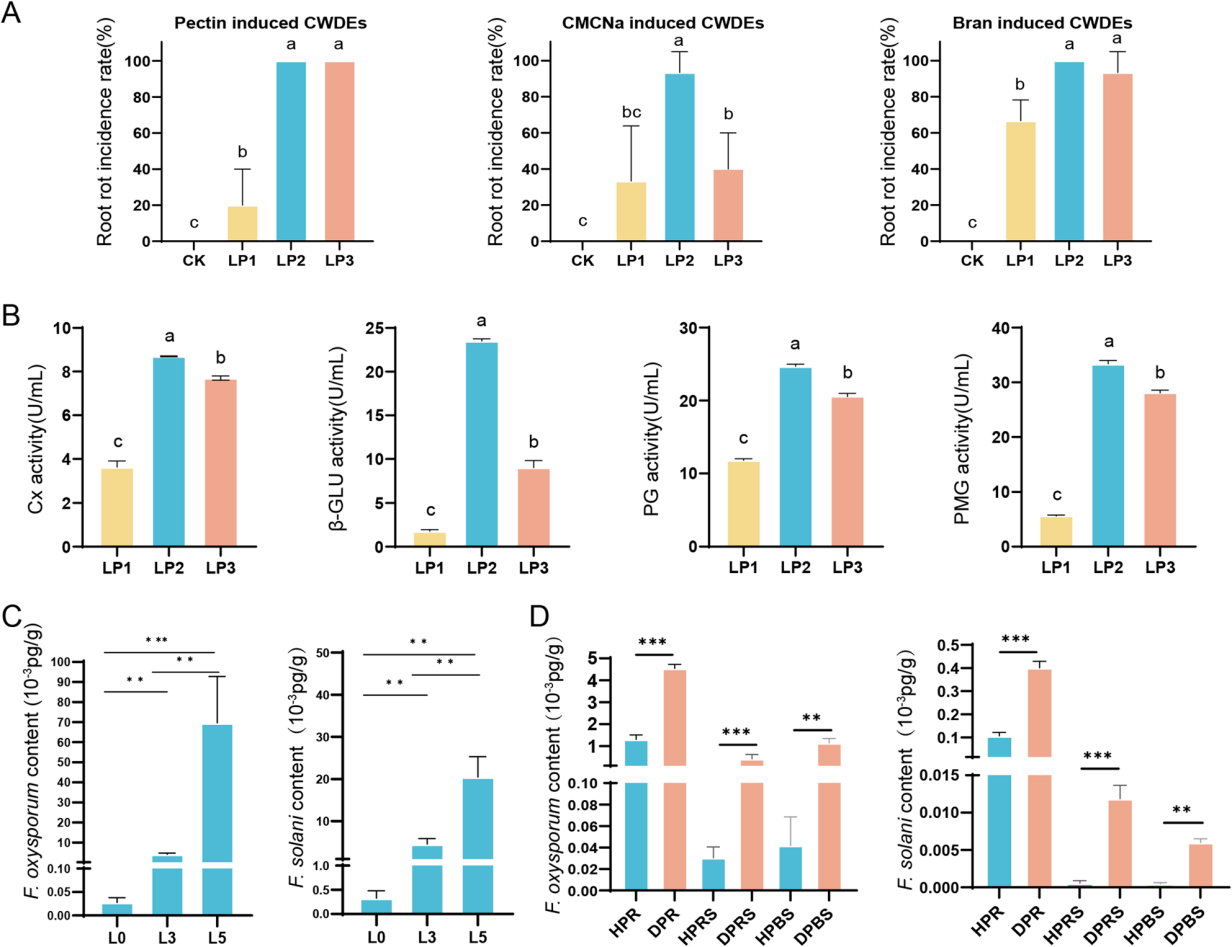
Assessment of *Fusarium* CWDE pathogenicity and biomass. **A** Effects of pectin, CMCNa, and bran-induced CWDEs on root rot incidence; **B** Comparison of the activities of carboxymethyl cellulase (Cx), β-glucosidase (β-GLU), polygalacturonase (PG), and polymethylgalacturonase (PMG); **C** Content of *F. solani* and *F. oxysporum* in *P. notoginseng* at different disease stages. L0: Healthy, asymptomatic; L3: Decay extended to the interior and is > 40% but ≤ 50%; L5: Decay extended to the interior, and is > 70%; **D ***F. solani* and *F. oxysporum* content in different niches: healthy *P. notoginseng* root tissue (HPR), diseased *P. notoginseng* root tissue (DPR), healthy *P. notoginseng* rhizosphere soil (HPRS), diseased *P. notoginseng* rhizosphere soil (DPRS), healthy *P. notoginseng* bulk soil (HPBR), diseased *P. notoginseng* bulk soil (DPBS). a, b, and c indicate significant differences (*P* < 0.05); two-sided Student’s *t*-test: *P < 0.05; **P < 0.01; ***P < 0.001

The mechanistic basis for virulence differences was elucidated through comparative analysis of cell wall-degrading enzyme (CWDE) profiles. Crude enzyme extracts derived from *F. solani* strain LP2 demonstrated greater tissue degradation capacity compared to those from *F. oxysporum* LP1 (Fig. 3A). Quantitative enzymatic assays revealed that LP2 and LP3 produced significantly higher activities of cellulase (Cx), β-glucosidase (β-GLU), polygalacturonase (PG), and polymethylgalacturonase (PMG) compared to LP1. None of the strains produced detectable PGTE or PMTE. The overall CWDE activity profile followed the order: LP2 > LP3 > LP1 (Fig. 3B; Fig. S3A-C), consistent with the observed virulence hierarchy (Fig. 3A).

However, RT-qPCR analysis revealed an inverse ecological pattern: *F. oxysporum* LP1 consistently accumulated greater biomass than the *F. solani* isolates (LP2/LP3) in both rhizosphere soil and infected root tissues (Fig. 3C, D). This pattern is consistent with microscopic observations showing a higher abundance of surface-associated spores in LP1-inoculated roots (Fig. 2B).

Overall, our study found an inverse relationship between the pathogenicity of *Fusarium* species and fungal biomass. *F. oxysporum* exhibited higher fungal biomass but weaker pathogenicity, while *F. solani* had lower biomass but stronger pathogenicity.

### Construction of a synthetic community to control *Fusarium* root rot

#### Construction and evaluation of synthetic microbial consortia

**Fig. 4:**
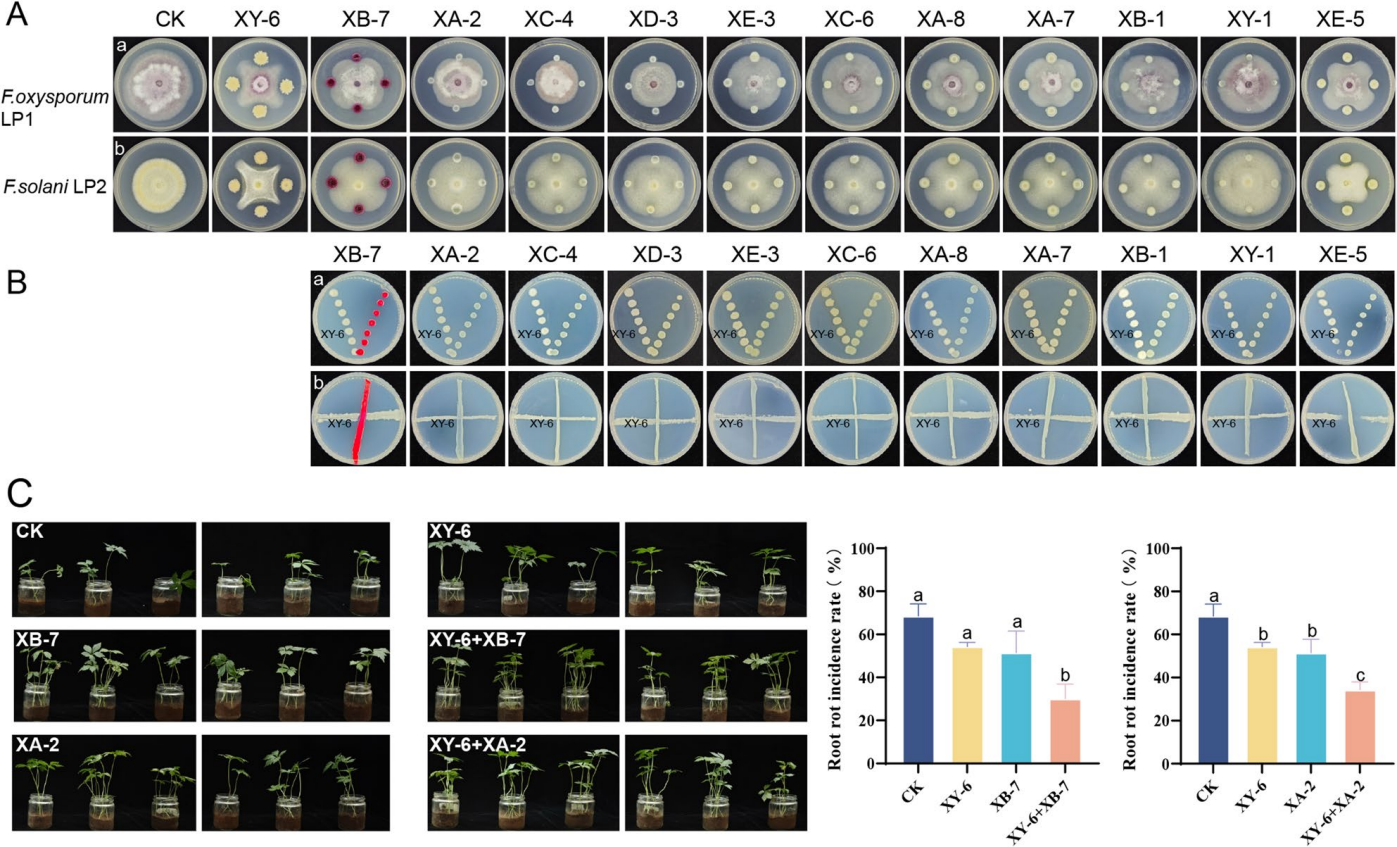
Antagonistic activity, compatibility, and root rot control evaluation of SynComs. **A** Effect of single strains on the growth of *F. oxysporum* LP1 and *F. solani* LP2 on PDA medium, *n* = 3; **B** Compatibility evaluation of XY-6 with 11 other candidate strains. In the V-shaped confrontation assay, XY-6 was placed on the left side of the plate and the candidate strain on the right; in the cross-streaking assay, XY-6 was in horizontal streaks and the candidate strain in vertical streaks, *n* = 3; **C** Effect of SynComs (XY-6 + XB-7 and XY-6 + XA-2) on root rot incidence in *P. notoginseng* (*n* = 6). For each treatment, six plants are shown as two assembled image parts, with three pots on the left and three pots on the right. The boundary between the two parts is indicated by a white dividing line. Different lowercase letters (a, b, and c) indicate significant differences at *P* < 0.05

To tackle the multifaceted challenges posed by *Fusarium* species, we systematically evaluated microorganisms for antifungal properties. Among the strains tested, XY-6 demonstrated the strongest inhibitory effects, with suppression rates of 49.36% and 33.33% against the growth of *F. oxysporum* LP1 and *F. solani* LP2, respectively. Morphological analysis preliminarily identified XY-6 as *Bacillus* (Fig. 4A; Supplementary Table 1). Through comprehensive compatibility testing (Fig. 4B), we successfully established ten distinct microbial consortia by combining XY-6 with compatible bacterial partners. Subsequent plant experiments from ten microbial consortia identified two particularly effective SynComs: the XY-6 + XB-7 pairing and XY-6 + XA-2 consortium. These microbial teams substantially improved seedling viability (96%–104% enhancement) and decreased root rot occurrence by 50%-56%, demonstrating superior performance compared to individual strain applications (which showed only 21%–25% disease reduction) (Fig. 4C, S4). Hemolysis assays confirmed the biosafety of all strains (Fig. S5), indicating their potential for practical application.

### The *Bacillus-Serratia* (BS) consortium controls *Fusarium* root rot through plant systemic resistance induction and direct antagonism

#### The BS consortium confers dual functionality—growth promotion and disease suppression

**Fig. 5:**
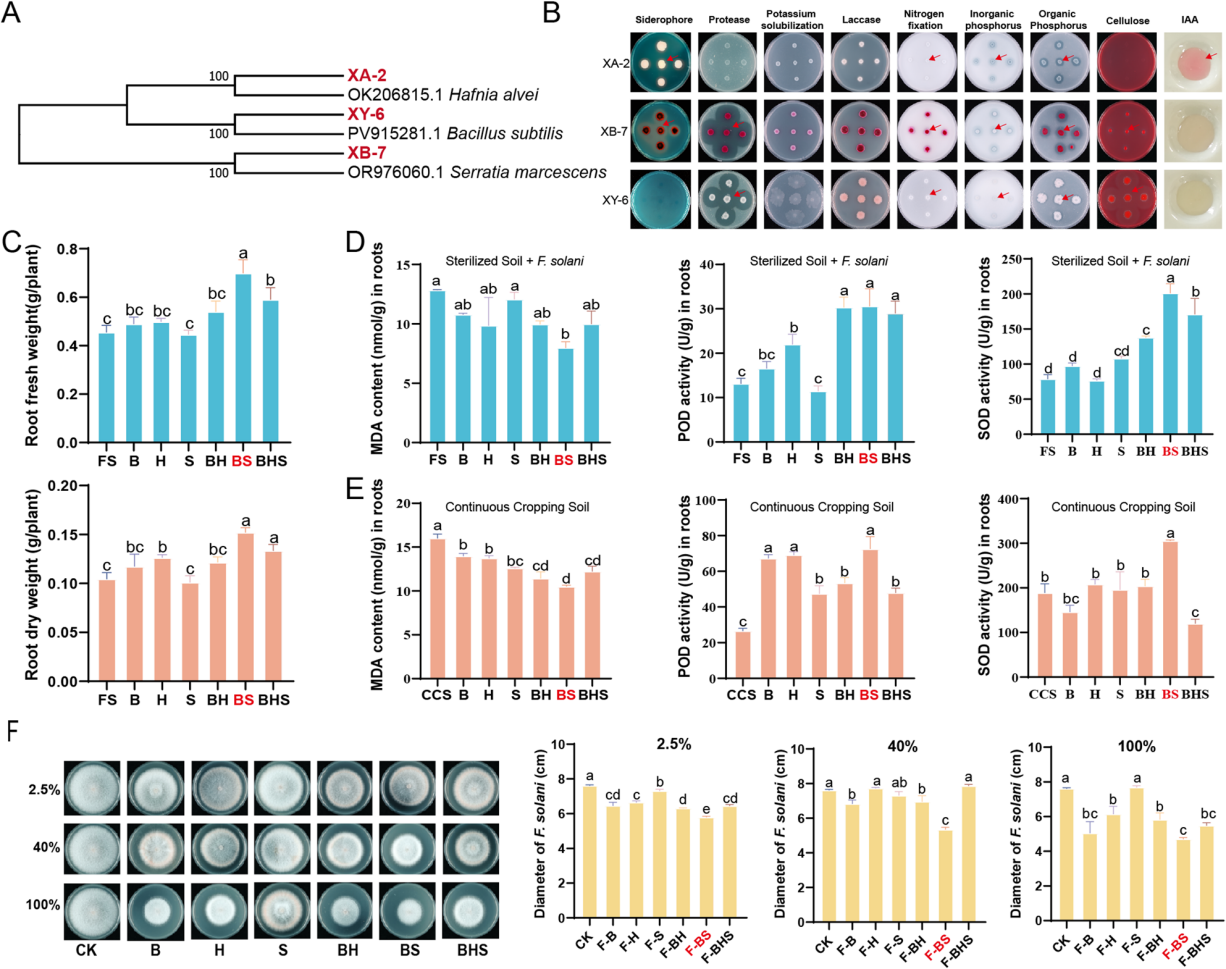
Identification of SynComs and evaluation of their growth-promoting, resistance-inducing, and *Fusarium-*antagonistic capabilities. **A** Phylogenetic tree of biocontrol bacteria; **B** Functional identification of XA-2, XB-7, and XY-6 for siderophore production, protease activity, potassium solubilization, laccase activity, nitrogen fixation, inorganic phosphorus solubilization, organic phosphorus solubilization, cellulose degradation, and IAA production; **C** Effect of SynComs BH, BS, and BHS on the fresh and dry weights of *P. notoginseng* roots; B, *B. subtilis* XY-6, S, *S. marcescens* XB-7, and H represents *H. alvei* XA-2. The experimental soil was autoclaved and then inoculated with *F. solani* LP2. The control group, designated as FS, refers to soil inoculated solely with *F. solani*; **D** Effect of SynComs on MDA content, POD, and SOD activities in roots of *P. notoginseng*; after soil sterilization, the spore suspension of *F. solani* LP2 was added, followed by the addition of SynComs; FS represents sterilized soil supplemented with *F. solani*, *n* = 3; **E** Effect of SynComs on MDA content, POD, and SOD activities in roots of *P. notoginseng*; the soil used was continuous cropping soil (CCS), *n* = 3; **F** Effect of sterile fermentation filtrates of SynComs at concentrations of 2.5%, 40%, and 100% on the growth diameter of *F. solani* LP2, *n* = 3; different letters indicate significant differences (*P* < 0.05)

Through comprehensive phylogenetic and functional analyses, three key biocontrol agents were identified: *Bacillus subtilis* XY-6 (B), *Serratia marcescens* XB-7 (S), and *Hafnia alvei* XA-2 (H). Among these, *S. marcescen*s XB-7 exhibited the broadest spectrum of plant growth-promoting traits, including nitrogen fixation, phosphate solubilization, protease production, cellulase activity, and siderophore biosynthesis (Fig. 5A, B). In plant assays, the BS consortium (*B. subtilis* XY-6 + *S. marcescens* XB-7) exhibited a unique dual functionality, significantly enhancing root biomass of *P. notoginseng* (by 54% and 46% in fresh and dry weights, respectively) while simultaneously reducing *F. solani*-induced root rot by 61% (Fig. 5C, S6). These protective effects likely arise from a coordinated, multi-component biological mechanism.

#### Synthetic consortium BS induces host systemic resistance and directly inhibits pathogen growth

The beneficial effects of the BS consortium were mediated through multiple biological processes. Within plant tissues, BS effectively activated systemic defense responses by enhancing antioxidant enzyme activities, thereby alleviating oxidative damage. Further analyses showed that BS significantly mitigated oxidative stress in *P. notoginseng* roots, most notably by reducing malondialdehyde (MDA) content—an indicator of membrane lipid peroxidation—by 38% and 34% in sterilized soil inoculated with *F. solani* and in continuous-cropping soils, respectively. Concurrently, BS markedly enhanced the activities of key antioxidant enzymes, with peroxidase (POD) activity increasing by 133% and 173%, and superoxide dismutase (SOD) activity increasing by 155% and 62% under the respective soil conditions (Fig. 4D-E). These results indicate that BS can induce plant resistance to *Fusarium* stress.

The disease-suppressive efficacy of BS was further supported by in vitro assays. Cell-free filtrates derived from the BS consortium exhibited the strongest inhibitory effects on *F. solani* LP2 mycelial growth, achieving inhibition rates of 24%, 30%, and 38% at concentrations of 2.5%, 40%, and 100%, respectively, which were consistently higher than those of individual strain filtrates (Fig. 5F). This enhanced antifungal activity is likely attributable to the synergistic action of non-volatile metabolites produced by the synthetic microbial community.

### The BS SynCom reshapes the soil microbiome to maintain plant health

#### Functional remodeling of the rhizosphere mycobiome

**Fig. 6:**
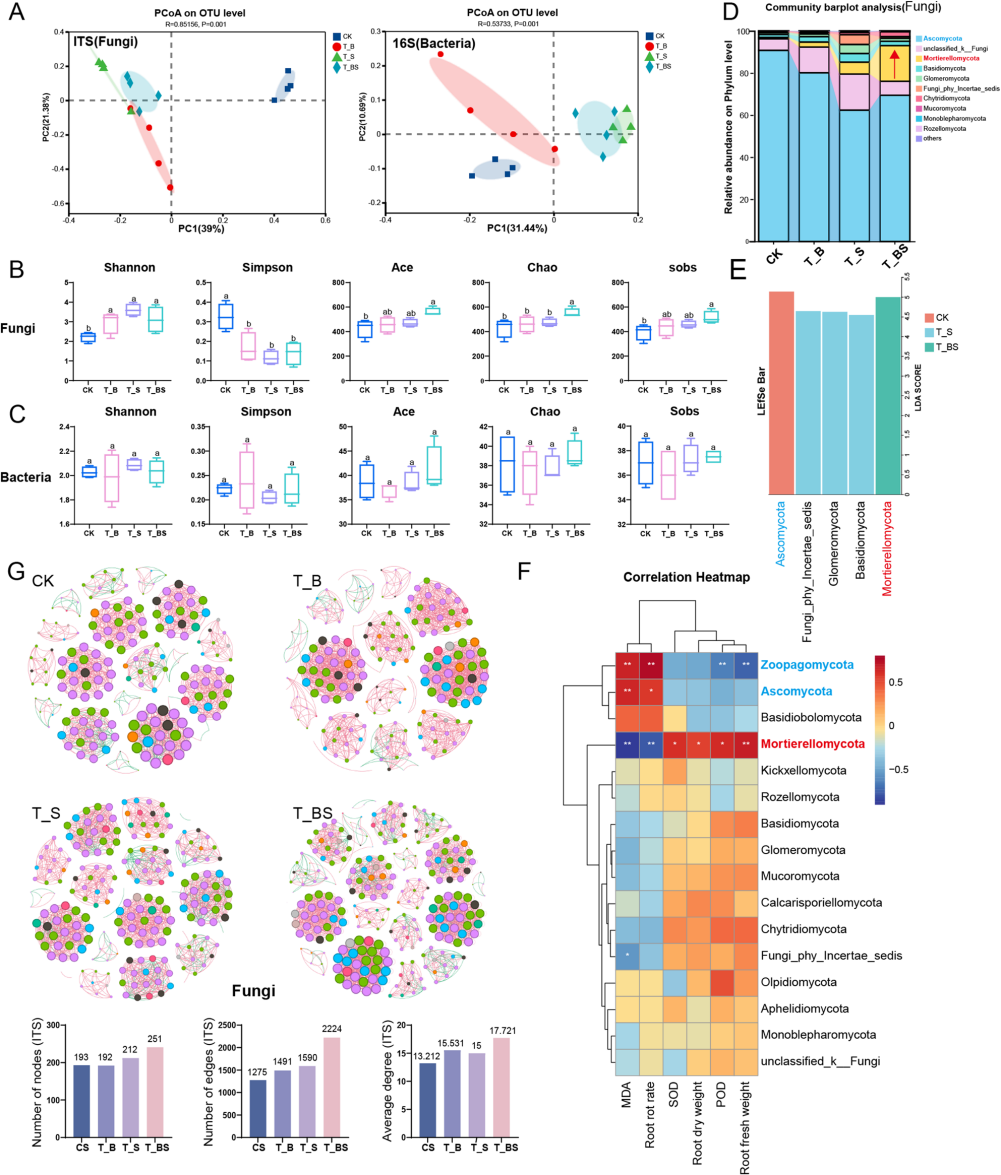
Effect of SynCom on the rhizosphere soil microecology. **A** PCoA of rhizosphere soil fungi and bacteria following treatment with SynCom and single strains, *n* = 4; **B**, **C** Effect of SynCom on soil fungal and bacterial α-diversity; different letters indicate significant differences (*P* < 0.05); **D** Differences in phylum-level composition of fungi; **E** Fungal phylum-level LEfSe analysis of core microbiota following SynComs treatment; **F** Correlation heatmap between phylum-level fungal relative abundance and *P.notoginseng* root rot incidence, root fresh/dry weight, SOD activity, POD activity, and MDA content. * indicates *P* < 0.05, ** indicates *P* < 0.01; **G** Fungal co-occurrence network diagram of SynCom-treated groups and statistics on node number, edge number, and average degree. CK: Water treatment; T_B: Treatment with *B. subtilis* XY-6; T_S: Treatment with *S. marcescens* XB-7; T_BS: Treatment with *B. subtilis* XY-6 and *S. marcescens* XB-7

The microbial community structure in the rhizosphere underwent substantial modifications following inoculation, with the BS consortium demonstrating particularly pronounced effects, as evidenced by both sequencing results (Fig. S7) and principal coordinate analysis (Fig. 6A). Notably, only the synthetic consortium BS significantly increased fungal α-diversity indices, including Ace, Chao, and Sobs (*P* < 0.05), while bacterial α-diversity remained relatively unaffected (Fig. 6B-C). This observation was further supported by OTU analysis, which revealed maximal richness in BS-treated samples (Fig. S8). The consortium induced profound functional alterations within the microbial assembly, most strikingly through a dramatic 24-fold amplification of Mortierellomycota, a beneficial fungal phylum that subsequently dominated the community. Concurrently, the abundance of Ascomycota, which includes various Fusarium species, was significantly reduced (Fig. 6D). LEfSe analysis further identified Mortierellomycota as the phylum with the highest LDA score in the BS treatment, whereas Ascomycota scored highest in the CK (Fig. 6E), indicating that Mortierellomycota became a core constituent of the fungal community following BS introduction.

Analysis of the 50 most abundant fungal genera showed that both single-strain inoculations and the SynCom BS significantly increased the relative abundance of *Mortierella*, a potentially beneficial genus within Mortierellomycota, consistent with phylum-level trends (Fig. S9A). At the bacterial genus level, strain B (*B. subtilis*) enriched *Bacillus*, while strain S (*S. marcescens*) increased *Serratia*. Notably, the BS consortium simultaneously enhanced both genera (Fig. S9B), indicating successful root colonization and synergistic establishment in the *P. notoginseng* rhizosphere.

Correlation analyses revealed strong positive associations between the relative abundance of Mortierellomycota and plant growth parameters (root fresh and dry weights) as well as defensive enzyme activities (POD and SOD), whereas root rot incidence and malondialdehyde (MDA) content were negatively correlated (Fig. 6F). These findings suggest that the BS SynCom may enhance plant health by modulating Mortierellomycota populations. Overall, shifts in the microbial community were significantly associated with improved plant health, with fungal community reorganization emerging as the dominant feature of this transformation.

#### BS Promotes the complexity and stability of microbial networks

Analysis of co-occurrence networks demonstrated that the BS consortium specifically improved the structural robustness and interconnectedness of fungal community networks (Fig. 6G). When compared with networks developed under individual strain applications, the BS fungal network showed significant enhancements across multiple parameters: edge quantity increased by 31%–49%, node quantity rose by 18%–31%, and average degree improved by 14%–18% (Fig. 6G). These results imply that the synthetic microbial community promoted more extensive and potentially cooperative fungal interactions. Regarding bacterial networks, all treatments led to increased network complexity. However, the BS treatment did not surpass the network complexity achieved by the single *S. marcescens* application (Fig. S10), indicating that *S. marcescens* XB-7 may play an important role in shaping bacterial network architecture. Together, these observations reveal that the BS consortium selectively strengthens the structural complexity and resilience of fungal interaction networks, thereby enhancing its biological control performance.

## Discussion

**Fig. 7:**
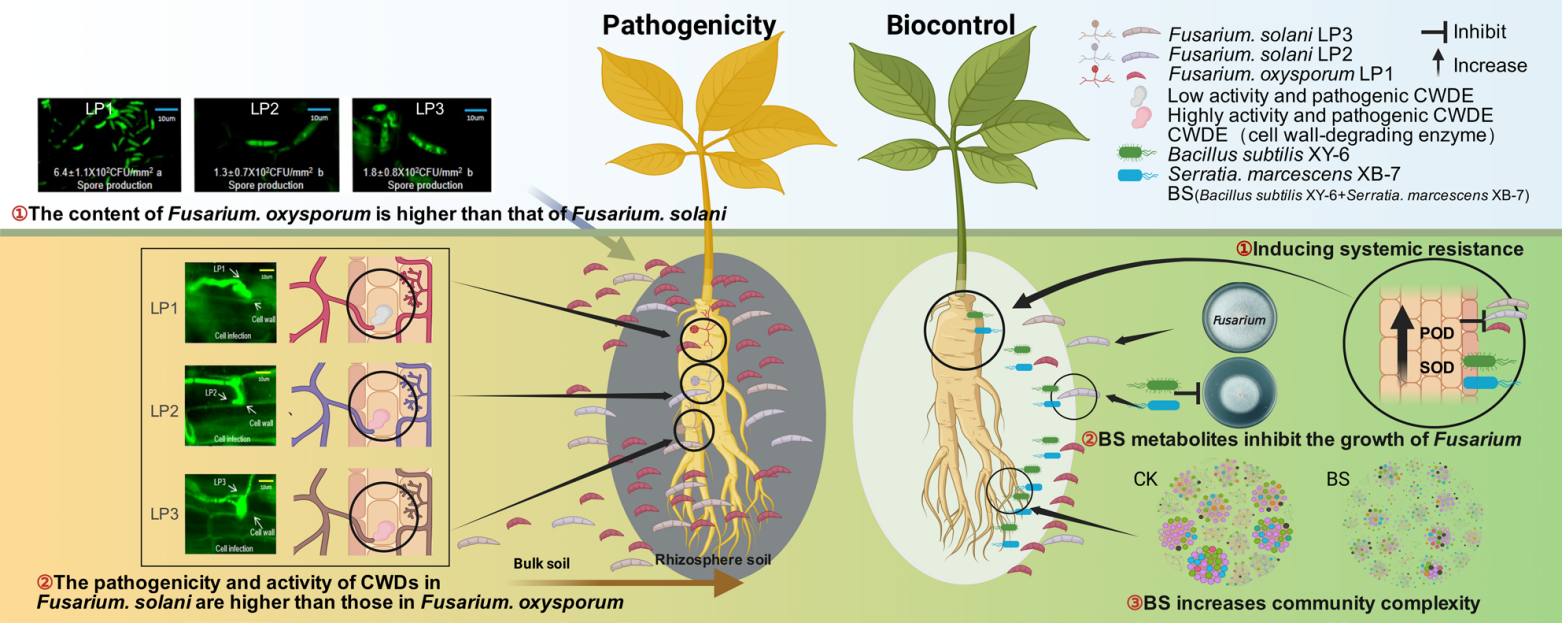
Schematic diagram illustrating the pathogenic and biocontrol mechanisms of *Fusarium* root rot in *P. notoginseng. *The left panel illustrates two pathogenic mechanisms: *Fusarium* spp. cause root rot in *P. notoginseng* via differences in population abundance and CWDE activity. The right panel shows three biocontrol mechanisms against *Fusarium* root rot: inducing plant systemic resistance, directly antagonizing *Fusarium* pathogens, and and improving the soil microbial community

This study bridges microbial ecology and synthetic community design. We first identified a fundamental divergence in pathogenic strategy within *Fusarium* species: *F. oxysporum* thrives via population expansion, whereas *F. solani* exhibits enhanced virulence. Building on this ecological insight, we engineered a novel *Bacillus-Serratia* (BS) consortium. This synthetic community effectively counters the dual threat through coordinated, multi-faceted interactions (Fig. 7).

### The engineered microbial consortium operates through combined direct inhibition and activation of plant defense mechanisms

The BS consortium demonstrated significant disease control through a dual mechanism involving direct pathogen inhibition and host immune activation. In laboratory tests, the combined culture filtrate from BS strains exhibited superior antifungal activity against *F. solani* compared to individual strain preparations (Fig. 5F), suggesting cooperative synthesis of antimicrobial compounds.

Evaluation of enzyme activities in *P. notoginseng* demonstrated that the SynCom BS significantly reduced MDA content and enhanced the activities of POD and SOD in sterilized soil infested with *F. solani*, outperforming both the control and individual strains. Similar results were reproduced in continuous cropping soil, indicating that BS may enhance resistance to *Fusarium* infection by inducing systemic defense responses (Fig. 5C-E). SOD, a key enzyme in plant stress response, plays a vital role in maintaining reactive oxygen species (ROS) homeostasis [45]. Studies have shown that enhanced activities of SOD can confer strong salt tolerance and root rot resistance in soybean [46]. Increased POD activity also serves as an indicator of induced systemic resistance [22]. MDA content is a widely used parameter for assessing lipid peroxidation in plant tissues [47], and reducing MDA levels can improve plant resilience [48]. Our results indicate that the SynCom BS enhances plant disease resistance by reducing MDA content—a key lipid peroxidation product—while boosting the activities of POD and SOD (Fig. 5D-E).

These complementary mechanisms—direct microbial antagonism coupled with plant defense stimulation—constitute the fundamental biological processes responsible for disease management.

### Long-term pathogen suppression is achieved through functional modification of the rhizosphere microbial community

The BS consortium significantly enhanced fungal α-diversity, particularly species richness, as indicated by higher Shannon, Ace, Chao, and Sobs indices, with Chao values exceeding those of single-strain treatments (Fig. 6B). Li et al. emphasized the role of microbial diversity in promoting ecosystem multifunctionality, including soil nutrient cycling and enzyme activities, but found no significant correlation between microbial α-diversity and plant growth response ratios [49]. In contrast, our findings show that the enhanced fungal α-diversity under BS treatment coincided with increased plant biomass and antioxidant capacity in *P. notoginseng*, suggesting a coordinated plant-fungal α-diversity interaction. Consistent with previous reports, bacterial α-diversity remained largely unchanged [49] (Fig. 6C), indicating that the BS consortium primarily reshaped the fungal community. Overall, these results suggest that a bacterial synthetic consortium can selectively enhance fungal α-diversity, providing new insights into the design of microbial consortia for plant-soil systems.

The altered microbial community composition correlates with improved physiological performance in both soil and plants. Our data reveal a significant relationship between increased Mortierellomycota populations and elevated activity of key plant defense enzymes (*P* < 0.05) (Fig. 6F). *Mortierella*, a core genus within Mortierellomycota, is known to enhance soil fertility and reduce root rot incidence in *P. notoginseng* [50, 51]. In combination with *Bacillus* and *Serratia* strains (Fig. S9B), these taxa likely act synergistically to boost rhizosphere metabolic and enzymatic functions. *Mortierella* interacts with bacteria to produce specialized metabolites, such as biosurfactants and antibiotics, which suppress pathogens [52], while also stimulating nutrient-mobilizing enzymes (e.g., phosphatases) that promote soil fertility and plant defense [53]. We propose that the BS consortium exemplifies a “multi-mechanism synergy” strategy, where *Bacillus* and *Serratia* antagonize pathogens and induce immunity, while *Mortierella* enriches soil ecosystem functions, offering a promising approach for next-generation biocontrol.

Co-occurrence network analysis identifies robust correlations among taxa, linking strongly associated species into modular structures [54]. Previous studies showed that soil infestation with *F. oxysporum* reduces key fungal network metrics—such as total edges, average degree, and average clustering coefficient—simplifying network architecture and decreasing seedling survival and biomass in *P. notoginseng* [50]. In contrast, the BS consortium significantly increased network complexity, as evidenced by higher node/edge counts and average degree compared to control and single-strain treatments. Notably, *S. marcescens* XB-7 alone also enhanced these topological metrics (Fig. 6G). Given the concurrent reduction in root rot incidence, we propose that *S. marcescens* XB-7 plays a critical role in promoting cooperative interactions and increasing network complexity within the consortium. These results suggest that BS reinforces microbial network architecture, potentially sustaining soil ecological stability and indirectly suppressing *Fusarium* infection in *P. notoginseng*.

### Pathogen strategy dichotomy informs precise biocontrol design

Our findings reveal a distinct functional dichotomy between *F. oxysporum* and *F. solani*. As shown in Fig. 3, interspecific differences in CWDE profiles largely explain the variation in pathogenicity, with *F. solani* exhibiting the highest virulence. In contrast, the superior sporulation capacity of *F. oxysporum* (Figs. 2 and 3) underpins its numerical dominance and competitive establishment. This trade-off between “high virulence” and “high abundance” highlights the limitations of broad-spectrum suppression and underscores the need for biocontrol strategies that specifically target pathogen-specific ecological functions. However, the molecular mechanisms underlying this ecological trade-off remain incompletely understood, and further studies are needed to clarify this functional divergence.

The synthetic consortium BS likely suppresses *Fusarium* infection in *P. notoginseng* through the combined reduction of pathogen density and virulence-associated enzyme activity. *Bacillus* spp. have been widely reported to decrease *Fusarium* populations in soil and plant tissues, leading to lower disease incidence across multiple cropping systems [55]. In parallel, chitinase-producing *S. marcescens* strains suppress *Fusarium* growth by degrading fungal cell walls, reducing pathogen virulence [56]. Beyond population-level suppression, biocontrol agents can directly attenuate fungal virulence by downregulating CWDE expression. Recent evidence shows that *Bacillus*-based treatments significantly repress genes encoding key hydrolytic enzymes, including pectin lyases and xylanases, thereby limiting host penetration and colonization by *F. oxysporum* [57]. Given the central role of CWDEs in fungal pathogenicity, their suppression provides a mechanistic explanation for reduced disease severity [58].

Overall, integrating targeted pathogen suppression with microbiome-mediated functional regulation provides an ecologically informed biocontrol strategy. By aligning microbial interventions with pathogen virulence mechanisms, this approach offers a precise and sustainable framework for managing complex soil-borne diseases.

## Conclusion

To summarize, the synthetic microbial consortium comprising *Bacillus* and *Serratia* (BS) demonstrates comprehensive suppression of *Fusarium* root rot through multiple coordinated mechanisms. These include direct microbial antagonism, activation of plant defense responses, and strategic modification of rhizosphere microbial communities to establish a protective and ecologically stable environment. This study contributes significant insights into pathogen dynamics within medicinal plant ecosystems while introducing an innovative microbial-based solution with synergistic effects for eco-friendly disease control. The proposed framework offers a multi-pronged approach to sustainable plant protection. Future research should focus on elucidating the underlying molecular mechanisms and translating these promising findings into robust field applications.

## Supplementary Information

Supplementary Material 1.

Supplementary Material 2.

## Data Availability

The nucleotide sequences generated in this study have been deposited in the NCBI GenBank database under the following accession numbers. For *F. oxysporum* strain LP1: LSU rRNA gene (PX678768), ITS region (PX674653), and translation elongation factor 1-α gene (TEF, PX700860). For *F. solani* strains: LP2 (LSU: PX678769; ITS: PX674654; TEF: PX700861) and LP3 (LSU: PX678770; ITS: PX674655; TEF: PX700862). Sequences for *H. alvei* XA-2, *B. subtilis* XY-6 and *S. marcescens* XB-7 are available under PX678125, PX678126, and PX678127, respectively. Raw soil microbiome sequencing data are accessible under BioProject accession PRJNA1379223 (https://www.ncbi.nlm.nih.gov/bioproject/PRJNA1379223).
