# Supplementary material for: Divergent pathogenic strategies of Fusarium species in Panax notoginseng and biocontrol by a Bacillus-Serratia consortium
Source: BMC Plant Biol. 2026 Mar 24;26:785. doi: 10.1186/s12870-026-08617-4 (PMC13134150; doi:10.1186/s12870-026-08617-4)
Supplement: Supplementary file 1 — Supplementary Material 1. [file 12870_2026_8617_MOESM1_ESM.docx]

**Supplementary Table 1.** Inhibition rate (%) of candidate strains against *Fusarium* spp.

| Strain number | *F.oxysporum* LP1 | *F.olani* LP2 |
| --- | --- | --- |
| XY-6 | 49.36±1.79a | 33.33±0.57a |
| XB-7 | 36.95±0.89bc | 20.46±0.57b |
| XA-2 | 33.07±1.19d | - |
| XC-4 | 35.66±0.78cd | - |
| XD-3 | 34.88±2.05cd | - |
| XE-3 | 22.48±0.00f | - |
| XC-6 | 20.93±1.55f | - |
| XA-8 | 37.21±0.78bc | - |
| XA-7 | 36.17±3.49bcd | 4.62±0.57c |
| XB-1 | 33.59±1.18cd | - |
| XY-1 | 28.17±4.27e | - |
| XE-5 | 39.53±1.56b | 33.66±4.32a |

Note: Lowercase letters in the table represent the significance (*P*<0.05) of the difference between different bacterial fluids treatments, respectively, and “-” represents that the candidate strains have no inhibition rate against pathogenic bacteria
