# Supplementary material for: Divergent pathogenic strategies of Fusarium species in Panax notoginseng and biocontrol by a Bacillus-Serratia consortium
Source: BMC Plant Biol. 2026 Mar 24;26:785. doi: 10.1186/s12870-026-08617-4 (PMC13134150; doi:10.1186/s12870-026-08617-4)
Supplement: Supplementary file 2 — Supplementary Material 2. [file 12870_2026_8617_MOESM2_ESM.docx]

***Supplementary Material***

**Supplementary Fig. 1.** *Fusarium* strains inoculated on *P. notoginseng*  without wounds in vitro.

**Supplementary Fig. 2.** Functional characterization of three *Fusarium* strains on different media. PSM: phosphorus solubilization medium (for phosphate solubilization); CAS: chrome azurol S agar (for siderophore production); CCM: carboxymethyl cellulose medium (for cellulolytic activity); LCM: laccase detection medium (for phenolic compound oxidation).

**Supplementary Fig. 3.** Standard curves and activity calculation of six *Fusarium* CWDEs. (A) Standard curve and enzyme activity calculation for PGTE and PMTE; (B) Standard curve and enzyme activity calculation for Cx and β-GLU; (C) Standard curve and enzyme activity calculation for PG and PMG.

**Supplementary Fig. 4.** SynComs reduce root rot and improve survival of *P. notoginseng*. (A) Images of *P. notoginseng* growth status after treatment with the SynComs; (B) Statistics of aboveground survival rate of *P. notoginseng*; (C) Statistics of root rot incidence of *P. notoginseng*. Different letters indicate significant statistical differences (*P* < 0.05).

**Supplementary Fig. 5.** Hemolysis test images of three bacterial strains.

**Supplementary Fig. 6.** SynCom effects on *P. notoginseng* root rot in *Fusarium*-treated soil. (A) Impact of SynComs on the incidence of *P. notoginseng* root rot in soil inoculated with *F. solani*. n = 6. Different letters denote statistically significant differences (*P* < 0.05). (B) Images of root rot symptoms under different treatments.

**Supplementary Fig. 7.** Rank-Abundance curves and Shannon rarefaction curves of fungal and bacterial communities. (A) Bacterial Rank-Abundance curves; (B) Fungal Rank-Abundance curves; (C) Bacterial Shannon rarefaction curves; (D) Fungal Shannon rarefaction curves.

**Supplementary Fig. 8.** Venn diagrams based on OTU numbers. (A) Bacterial Venn diagrams; (B) Fungal Venn diagrams. CK: Water treatment; T_B: Treatment with single bacterial suspension of *B. subtilis* XY-6; T_S: Treatment with single bacterial suspension of *S. marcescens* XB-7; T_BS: Treatment with SynCom of *B. subtilis* XY-6 and *S. marcescens* XB-7.

**Supplementary Fig. 9.** Heatmaps of top 50 microbes after *B. subtilis*, *S. marcescens*, and SynCom treatments. (A) Bacterial heatmaps; (B) Fungal heatmaps.

**Supplementary Fig. 10.** Bacterial co-occurrence network and topology after SynCom treatment. (A) Bacterial co-occurrence network analysis and statistics. CK: Water treatment; T_B: Treatment with single bacterial suspension of *B. subtilis* XY-6; T_S: Treatment with single bacterial suspension of *S. marcescens* XB-7; T_BS: Treatment with SynCom of *B. subtilis* XY-6 and *S. marcescens* XB-7.
